# I. Infectious Pneumonia. II. Tetanus. III. Diabetic Symptoms from Contaminated Drinking-water

**Published:** 1897-08

**Authors:** J. W. Petty

**Affiliations:** Greensboro, N. C.


### REPORTS OF CASES.

---

### I. INFECTIOUS PNEUMONIA. II. TETANUS. III. DIABETIC SYMPTOMS FROM CONTAMINATED DRINKING-WATER.

By J. W. Petty, D.V.S.,
GREENSBORO, N. C.

I. On March 26th I was sent by the Greensboro, North Carolina, Board of Agriculture to investigate an outbreak at Summerfield, which proved to be infectious pneumonia. Post-mortem was held on one gelding that had died, and the lungs showed circumscribed cavities, some of which contained gangrenous fluid; others contained pus and some broken-down lung-tissue, all of which showed the different stages of the disease and the progress made upon the different parts of the lungs. After a detailed examination I diagnosticated it as above. All the animals that had been sick for ten days died. Three horses at this time were in the congestive stage, and two showed elevated temperature, cough, loss of appetite, and restlessness, and some profuse diarrhœa and others constipation.

**The railroad trip alone would reward you for the time and expense.**

All the sick and exposed animals were immediately quarantined, and the sick treated with acetanilid as required to control fever, ammonia muriate, cinchona in whiskey, and other stimulants as indicated, and proper attention paid to the regulation of the bowels, ventilation, and clothing. The exposed ones were isolated and treated with prophylactics, and only one of them contracted the disease. All made a good recovery with the exception of slight abdominal breathing as a result of the cavities in the lungs.

II. I recently treated successfully two cases of tetanus with quinine in ℥ss doses three times a day, with belladonna and chloroform as needed. Horses with tetanus seem to stand a large amount of quinine.

III. Called recently to investigate a supposed contagious disease, I found ten horses in one stable showing symptoms of diabetes —*i. e.*, wasting of flesh and diuresis. All the patients urinated several times daily. Investigation showed that a quantity of pine sawdust used for bedding had drained into a shallow well, only a few yards away (down hill), from which the horses were watered. The turpentine, etc., could be tasted in the water, and it was very evident that the absorption from the pine-dust was the cause of the trouble. Of course, I ordered water from another well, and the horses are now free from the symptoms.

